# Maternal Interleukin-17 and disease activity influence pregnancy outcomes in women with psoriatic arthritis and ankylosing spondylitis

**DOI:** 10.1186/s12884-023-05364-4

**Published:** 2023-01-17

**Authors:** Ehab F. Girbash, Shaimaa M. Abdelwahab, Rehab M. Atef, Hala E. Sherif, Hussein M. Abdeldayem, Doaa S. Atta, Ahmed M. Radwan

**Affiliations:** 1 Obstetrics and Gynecology department, Faculty of Medicine, Zagazig University, Zagazig City, Egypt; 2 Rheumatology and Rehabilitation department, Faculty of Medicine, Zagazig University, Zagazig City, Egypt; 3 Clinical and Chemical Pathology department, Faculty of Medicine, Zagazig University, Zagazig City, Egypt

**Keywords:** Psoriatic arthritis, Ankylosing spondylitis, Maternal interleukin-17, Pregnancy outcomes

## Abstract

**Objective:**

We aimed in this study to evaluate the impact of maternal interleukin -17A and the activity of the illness on pregnancy outcomes in Psoriatic arthritis (PsA) and ankylosing spondylitis (AS) patients.

**Methods:**

This prospective cohort research was carried out on 48 Psoriatic arthritis and ankylosing spondylitis pregnant women attending the inpatient and outpatient clinics of the Rheumatology & Rehabilitation and Obstetrics & Gynecology Departments, Faculty of Medicine, Zagazig University Hospitals in Egypt and 30 apparently healthy age- and sex-matched pregnant women between January 1,2018, and December 31, 2019.

**Results:**

The study group patients had a higher risk of preterm labour (32–36 weeks gestation) (aRR 1.80, 95% CI 0.79–4.17), oligohydramnios (aRR 3.15, 95% CI 1.26–8.42), Caesarean delivery (aRR 1.57, 95% CI 1.41–2.68), and delivering infants small for gestational age (aRR 7.04, 95% CI 2.36–12.42). There was significant difference between the control group and the study groups regarding the level of IL-17A.

**Conclusion:**

Many females with PsA and AS have uninhibited pregnancy with regard to adverse events, but in comparison with normal pregnancies particularly with high IL-17A during the third trimester we noticed a growing risk of preterm labour, oligohydramnios and cesarean section. Further studies are needed to evaluate high maternal IL-17A levels and fetal outcomes.

## Background

Psoriatic arthritis (PsA) and ankylosing spondylitis (AS) are chronic inflammatory disorders that affect males and females at younger ages than other rheumatic diseases. Women with PsA and AS usually have normal pregnancy outcomes, although high disease activity during pregnancy may increase adverse pregnancy outcomes, according to 2004–2018 data from the Organization of Teratology Information Specialists (OTIS) Autoimmune Disease Project [1].

Psoriatic arthritis typically has varied joint and skin forms and variable degrees of severity and is a linked set of diseases called spondylarthritis [2]. Ankylosing spondylitis disease characterized by pain and reduced axial bone flexibility and peripheral arthritic, extra-articular symptoms or enthesitis, all of which can lead to limitations of activity, disability, or poor quality of life. Management involves physiotherapy and medication with nonsteroidal anti-inflammatory medicines (NSAIDs), inhibitors of tumor necrosis factor (TNF) and disease-modifying antirheumatic drugs (DMARDs). All of these variables can lead to poor pregnancy results for women [3]. Many reports have indicated that immune responses mediated by interleukin 17A (IL-17A) play a significant role in both diseases. The substantial clinical effectiveness seen in IL-17A inhibitors in the treatment of SpA and PsA is the best demonstration. However, there are many defects in understanding the effect of IL-17A on the pathophysiology of spondylarthritis, bone erosion, and bone development, and, an explanation for the uneven effect of IL-17A inhibition was identified. Registered studies in Norway cohorts have shown lower fertility in women with chronic arthritis and poor birth outcomes such as intrauterine growth retardation, preterm labour and Caesarean delivery [4]. The majority of ankylosing spondylitis patients from 6 weeks to 6 months after birth, developed an exacerbation of this condition. Limited studies have been carried out in prenatal and neonatal AS patients with a relatively small number of patients [5].

Overall, RA research reveals elevated risks of preterm birth, small-for-gestational-age (SGA), pre-eclampsia and caesarean deliveries from an overall perspective of chronic arthritis and pregnancy results [6]. AS studies and pregnancy outcomes are sparse however the latest research indicated that preterm birth and caesarean delivery chances had been elevated [7, 8].

We aimed in this study to evaluate the impact of maternal interleukin -17A and the activity of the illness on pregnancy outcomes in AS and PsA patients.

## Subjects and methods

This prospective cohort study involved 48 pregnant women with psoriatic arthritis and ankylosing spondylitis attending the inpatient and outpatient clinics of Rheumatology & Rehabilitation and Obstetrics & Gynecology Departments, Faculty of Medicine, Zagazig University Hospital in Egypt and 30 apparently healthy age- and sex-matched pregnant women between January 12,018, and December 31, 2019.

**Fig. 1:**
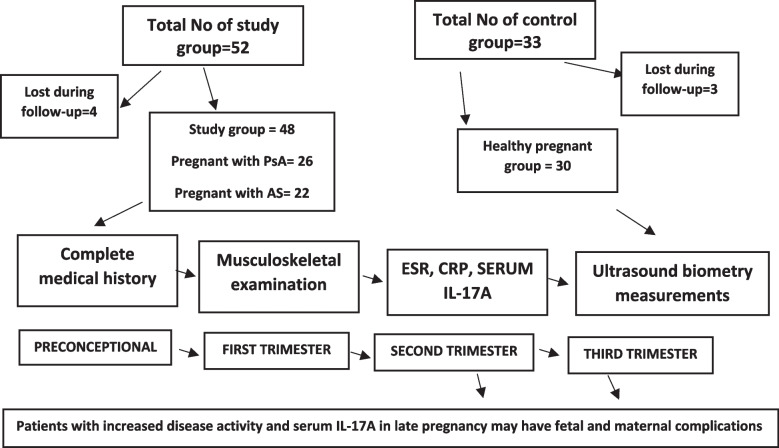
Flow chart of studied patients

All patients enrolled in this study provided informed consent before joining our study and all had the right to take away from the study without any interruption of their treatment plan and rights. All personal data of our enrolled patients were preserved and kept away from data retrieving personnel. A detailed flowchart (Fig. 1) was made for study demonstration.

The study group patients were diagnosed using modified New York criteria for ankylosing spondylitis [9] and classification for psoriatic arthritis (CASPAR) criteria [10].

Information was gathered in advance preconception (3 months to 1 year), throughout the 1st trimester (8–12 weeks), 2nd trimester (16–24 weeks) and 3rd trimester (28–34 weeks of pregnancy).

All pregnant women who consented to participate were enrolled at the 4 time points:

Complete medical history, medication exposures during pregnancy, obstetric history including maternal age, expected date of delivery, gravity, parity, gestational age at enrollment and at labour, pregnancy by ICSI, and previous preterm delivery or intrauterine growth retardation., family history, preconception body mass index (BMI), and socioeconomic status. Medication included start and end dates, indications, variations in dose and frequency, use of caffeine, nutritional supplements, folic acid intakes, infections, or antenatal investigation or other medical intervention. Any woman with other autoimmune diseases or other chronic diseases was excluded from the study.

General and local musculoskeletal examination.

Laboratory investigations.

The erythrocyte sedimentation rate (ESR) was determined by the Westergren method, the C-reactive protein (CRP) level was determined by the latex agglutination test, and HLA B27 (ELISA Kit) was estimated by collecting blood samples which stored at − 20 °C until tested for human HLA B27 using the Sunlong Biotech kit, China (catalog number: SL1056Hu).

IL-17A ELISA: ELISA is an enzyme-linked immunosorbent assay for the quantitative detection of human IL-17A. The kit was supplied by IBL International GmbH (Flughafenstr. 52A, 22,335 Hamburg, Germany).

Disease activity.

Using patient-reported assessments, including the Health Assessment Questionnaire (HAQ) [11] on a scale from 0 to 3, as well as pain score and patient global disease activity assessment on a scale from 0 to 100, the study group was evaluated at the same obstetric evaluation time points (pre-conceptional, first trimester, and third trimester). Next, the total pain score was divided by 10 for a range of 0–10, and the patient global assessment was performed in the same way. Cumulative Routine Assessment of Patient Index Data 3 (RAPID3) had three markers of disease activity and each marker was combined to yield a RAPID3 score ranging from 1 to 30, with active disease defined as a RAPID3 score ≥ 7 [12]. Active disease was defined as a HAQ score greater than 0.5.

Measures of pregnancy outcomes:

Premature birth (delivery before 37 weeks of gestation), small for gestational age (SGA—fetal weight is projected to be smaller than the 10th percentile for the child’s gestational age and sex), and delivery methods (vaginal or cesarean).

Inclusion criteria included a singleton pregnancy aged ≥18 years, with diagnosis of AS or PsA for at least 6 months, without major fetal anomalies, known chromosomal abnormalities or other autoimmune diseases, and gestational age of 28–40 weeks. Patients with a history of preterm labour, or any medical disorder were excluded from the study.

Ultrasound biometry measurements such as the biparietal diameter (BPD), head circumference (HC), abdominal circumference (AC), and femur length (FL) were taken on each fetus. Hadlock’s formula gives the estimated fetal weight (EFW). William’s tables were used to obtain the overall EFW percentile. The tables determine the birth weight percentile by gestational age and sex, and they are from a large population-based study with a sample size large enough to calculate the percentile in question [13, 14].

### Statistical analysis

Data collected throughout history, basic clinical examination, laboratory investigations and outcome measures were coded, entered and analyzed using Microsoft Excel software. The data collected were tabulated and analyzed by SPSS (statistical package for social science) version 25 (IBM, Armonk, NY, USA) on an IBM compatible computer.

Descriptive statistics were calculated for the data in the form of the mean and standard deviation± SD for quantitative data, and the frequency and distribution for qualitative data.

Regarding analytical statistics, in the statistical comparison between the different groups, the significance of differences was tested using one of the following tests after establishing their non normality by the Shapiro–Wilk test of normality: (1) Student’s t-test was used to compare the mean of two groups of quantitative data of parametric data. (2) the Mann-Whitney test (U test) was used to assess the statistical significance of the difference of a nonparametric variable between two study groups. (3) Intergroup comparison of categorical data was performed by using the chi square test (X2-value) and Fisher’s exact test (FET), and (4) Poisson regression yielded adjusted odds ratios with 95% confidence intervals. Poisson regression is used to predict a count outcome. These tests (Shapiro–Wilk test, Student’s t-test, and the Mann-Whitney test) help in demonstration of increased maternal and fetal risk in late pregnancy with increased disease activity and high IL-17 levels. A *P* value < 0.05 was considered statistically significant and > 0.05 statistically insignificant, while a P value < 0.01 was considered highly significant in all analyses.

## Results

The maternal age mean ± SD was 30.21 ± 2.13, 33.42 ± 5.11, and 31.34 ± 3.15 weeks in the control, psoriatic arthritis, and ankylosing spondylitis groups respectively. The mean ± SD of BMI (kg/m2) was 23.25 ± 4.17, 38.21 ± 3.15, and 24.81 ± 5.13 respectively. The maternal weight gain (kg) mean ± SD was 23.25 ± 4.17, 38.21 ± 3.15, and 24.81 ± 5.13 respectively. The socioeconomic score N (%) was 25 (83.3%), 24 (92.3%), and 20 (90.9%) respectively. There was a statistically significant increase in the Psoriatic Arthritis patients compared with the healthy group with regard to age, BMI and Socioeconomic Score (Table 1).

**Table 1:** Demographic data of studied groups

	Control (***N*** = 30)	PsA (***N*** = 26)	AS (***N*** = 22)	***P*** value
**Maternal age**_mean ± SD_	30.21 ± 2.13	33.42 ± 5.11	31.34 ± 3.15	P1 **< 0.001**
				P2 = 0.281
**Maternal BMI (kg/m2)**_mean ± SD_	23.25 ± 4.17	38.21 ± 3.15	24.81 ± 5.13	P1 **< 0.001**
				P2 = 0.462
**Maternal weight gain (kg)**_mean ± SD_	13.41 ± 2.25	12.37 ± 7.43	13.62 ± 5.15	P1 **=** 0.384
				P2 = 0.627
**Socioeconomic Score**_**N (%)**_ (High score = 1–3)	25 (83.3%)	24 (92.3%)	20 (90.9%)	P1 **= 0.015**
				P2 = **0.002**

*PsA* Psoriatic Arthritis, *AS* Ankylosing Spondylitis

*P1* control group vs PsA group, *P2* control group vs AS group

Regarding parity, primigravida N (%), and multigravida N (%), they were significantly higher in Ankylosing Spondylitis group 17 (77.2) and 14 (63.6) than in control groups 14 (46.6) and 9 (30). Abortion N (%) was significantly lower in Psoriatic Arthritis group 2 (7.6%) than in control group 5 (16.7%). Regarding a history of preterm labour N (%), gestational diabetes N (%) and preeclampsia N (%) there was no significant difference between the control group and the psoriatic arthritis, and the ankylosing spondylitis groups (Table 2).

**Table 2:** Comparison between groups regarding obstetric history

	Control (***N*** = 30)	PsA (***N*** = 26)	AS (***N*** = 22)	***P*** value
**Obstetric history**				
**Parity**				
Primigravida _**N (%)**_	14 (46.6)	11 (42.3)	17 (77.2)	P1 = 0.541, P2 = **0.002**
Multigravida _**N (%)**_	9 (30)	7 (26.9)	14 (63.6)	P1 = 0.832, P2 **< 0.001**
**Abortion**_**N (%)**_	5 (16.7)	2 (7.6)	5 (22.7)	P1 = **0.003**, P2 = 0.283
**Preterm labor**_**N (%)**_	6 (20)	4 (15.3)	6 (27.2)	P1 = 0.842, P2 = 0.735
**Gestational Diabetes**_**N (%)**_	2 (6.6)	1 (3.8)	2 (9)	P1 = 0.427, P2 = 0.371
**Preeclampsia**_**N (%)**_	1 (3)	3 (11.5)	2 (9)	P1 = 0.175, P2 = 0.281

M (SD) mean (standard deviation) and *p* value < 0.05 is significant

The mean ± SD of Disease duration (years) was 5.32 ± 5.61, and 6.37 ± 4.15 in the psoriatic arthritis, and ankylosing spondylitis groups respectively. Additionally, HLA B27 positive testing was higher in the PsA group than in the AS group. The disease was active according to Routine Assessment Patient Index Data with 3 measures in 10 (38.5%) and 9 (41%) patients, and according to the Health Assessment Questionnaire the disease was active in 4 (15.4%) and 6 (27.2%) patients in the PsA, and AS groups respectively. Regarding the medications usage distribution, in the psoriatic arthritis group 21 (80.7%) were using biologic DMARDs, 3 (11.5%) were using DMARDs and 10 (38.4%) were using NSAIDs, while in the ankylosing spondylitis group, 18(81.8%) were using biologic DMARDs, 6 (27.2%) were using DMARDs and 8 (36.3%) were using NSAIDs (Table 3).

**Table 3:** History Comparison between study groups regarding disease duration, activity and medication

	PsA (***N*** = 26)	AS (***N*** = 22)	***P*** value
**Duration (year)**_mean ± SD_	5.32 ± 5.61	6.37 ± 4.15	0.735
**HLA B27 positive testing**_*N* (%)_	19 (73.1)	16 (72.7)	0.428
**Active disease**			
RAPID 3 score _*N* (%)_	10 (38.5)	9 (41)	0.625
HAQ score _*N* (%)_	4 (15.4)	6 (27.2)	
**Medications**			
Biologic DMARDs _*N* (%)_	21 (80.7)	18(81.8)	0.263
DMARDs _*N* (%)_	3 (11.5)	6 (27.2)	
NSAIDs _*N* (%)_	10 (38.4)	8 (36.3)	
Corticosteroids _*N* (%)_	11 (42.3)	8 (36.3)	

*HAQ* Health Assessment Questionnaire, *RAPID 3* Routine Assessment Patient Index Data with 3 measures, *DMARDs* Disease-Modifying Anti-Rheumatic Drugs, *NSAIDs* Non-Steroidal Anti-Inflammatory Drugs. Active disease when HAQ score > 0.5 and RAPID3 score ≥ 7

**Fig. 2:**
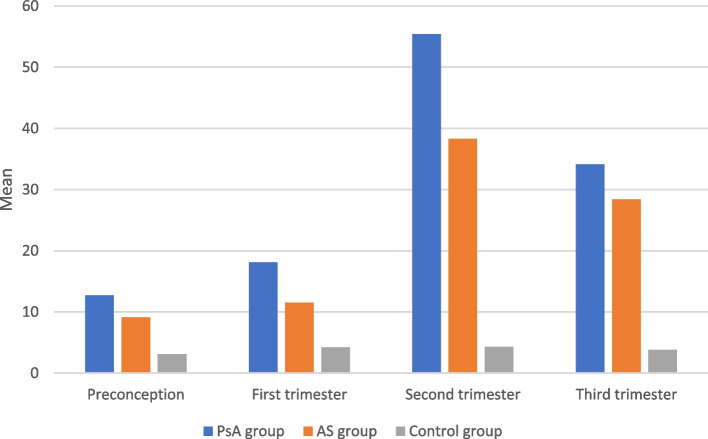
Comparison between study and control groups regarding IL-17

There was a significant difference between the control group and the psoriatic arthritis, and the ankylosing spondylitis groups at preconception, and the 1st,2nd and 3rd trimesters regarding CRP and IL-17A (Fig. 2) while there was no statistically significant difference between preconception, and the 1st,2nd and 3rd trimesters with regard to CRP and IL-17A (Table 4).

**Table 4:** Comparison between PsA and AS groups regarding laboratory investigations and disease activity at time points of study

	Preconception M (SD)	First trimester M (SD)	Second trimester M (SD)	Third trimester M (SD)	***P*** value
**CRP (mg/L)**					
**PsA group**	6.1 ± 2.1	6.9 ± 2.6	10.9 ± 4.7	9.3 ± 3.2	0.371
**AS group**	8.4 ± 3.3	9.1 ± 3.8	15.2 ± 5.3	15.9 ± 5.5	0.526
**Control group**	1.1 ± 0.6	1.3 ± 1.2	1.9 ± 1.3	2.3 ± 1.4	0.152
***P*** **value**	P1 = 0.026*	P1 = 0.015*	P1 = 0.001*	P1 = 0.001*	
	P2 = 0.001*	P2 = 0.001*	P2 = 0.001*	P2 = 0.001*	
**IL-17A (pg/L)**					
**PsA group**	12.7 ± 2.4	18.1 ± 12.1	55.4 ± 31.1	34.1 ± 18.1	0.073
**AS group**	9.1 ± 4.3	11.5 ± 4.9	38.3 ± 24.3	28.4 ± 15.6	0.126
**Control group**	3.1 ± 2.1	4.2 ± 2.3	4.3 ± 2.6	3.8 ± 2.2	0.635
***P*** **value**	P1 = 0.001*	P1 = 0.001*	P1 = 0.001*	P1 = 0.001*	
	P2 = 0.001*	P2 = 0.001*	P2 = 0.001*	P2 = 0.001*	
**Disease Activity**					
**RAPID 3 score**					
< 7	46 (95.8)	45 (73.8)	32 (66.7)	40 (83.3)	0.625
≥ 7	2 (4.2)	3 (6.2)	16 (33.3)	8 (16.7)	0.152
**HAQ score**					
0–1	44 (91.7)	43 (89.6)	35 (72.9)	37 (77.1)	0.471
1.1–3	4 (8.3)	5 (10.4)	13 (27.1)	11 (22.9)	0.138

*RAPID3* Routine Assessment of Patient Index Data 3, *HAQ* Health Assessment Questionnaire, *IL-17A* interleukin 17A, *CRP* C-reactive protein, *M (SD)* mean (standard deviation)

**Fig. 3:**
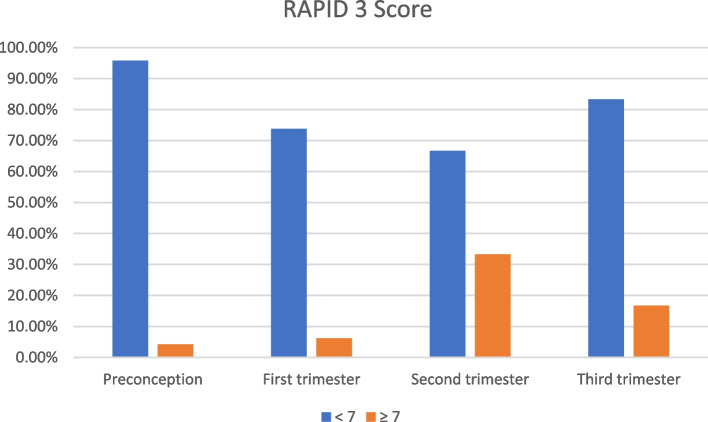
RAPID 3 Score in study groups

**Fig. 4:**
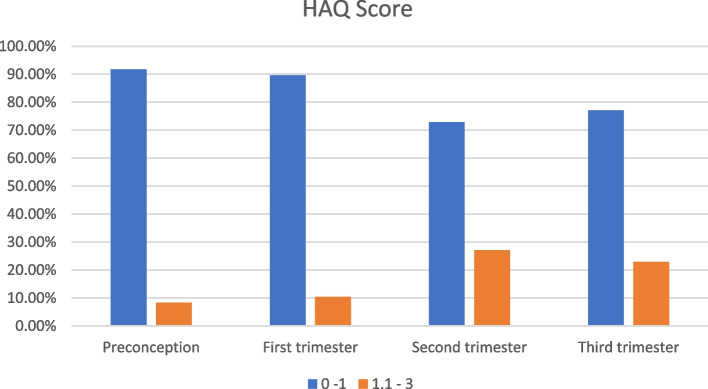
HAQ Score in study groups

Additionally, there was no significant difference between the control and study groups regarding the RAPID 3 score and HAQ score (Figs. 3 & 4) in the preconception, 1st,2nd and 3rd trimesters (Table 4).

**Fig. 5:**
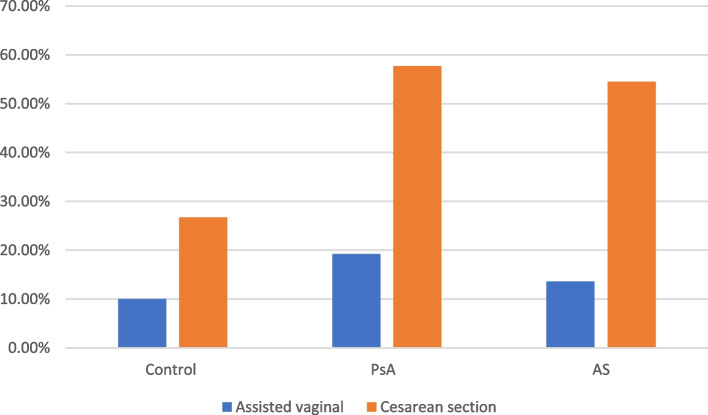
Comparison between study and control groups regarding Delivery

Comparing to healthy controls, the Women in the study groups had a higher risk of preterm labour (32–36 weeks gestation) (aRR 1.80, 95% CI 0.79–4.17), oligohydramnios (aRR 3.15, 95% CI 1.26–8.42), and cesarean delivery (Fig. 5) (aRR 1.57, 95% CI 1.41–2.68) and a higher risk of SGA fetuses (aRR 7.04, 95% CI 2.36–12.42) (Table 5). The Caesarean delivery indication was related only to failed labour progress with early fetal distress without any maternal pelvic abnormality or fetal malposition or malpresentation.

**Table 5:** Comparison between groups regarding pregnancy outcome

	Control (***N*** = 30)	PsA (***N*** = 26)	AS (***N***==22)	Adjusted OR (95% CI)	
	***N*** (%)	***N*** (%)	***N*** (%)	a OR * 1	a OR * 2
***Gestational age at delivery:***					
**Preterm < 37 weeks**_N (%)_	3 (10)	7 (26.9)	4 (18.2)	**1.80(0.79–4.17)**	1.48 (0.65–3.38)
**Term ≥ 37 weeks**_N (%)_	27 (90)	18 (69.2)	16 (72.7)	1.32 (0.57–1.76)	1.01 (1.48–2.36)
***Obstetric complications:***					
**Pre-eclampsia**					
Early onset (< 34 wk.) _N (%)_	0	3 (11.5)	1 (4.5)	N/A	N/A
Late onset (≥ 34 wk.) _N (%)_	3 (10)	5 (19.2)	2 (9)	2.01 (0.81–2.35)	0.93 (0.46–1.42)
**Gestational diabetes**_N (%)_	2 (6.6)	3 (11.5)	1 (4.5)	1.24 (0.51–1.63)	0.62 (0.35–2.21)
**Oligohydramnios**_N (%)_	3 (10)	7 (26.9)	4 (18.2)	**3.15 (1.26–8.42)**	2.14 (0.26–10.43)
**APH**_N (%)_	0	1 (3.8)	1 (4.5)	N/A	N/A
**PPROM**_N (%)_	1 (3.3)	3 (11.5)	2 (9)	1.34(0.73–2.49)	0.95 (0.35–1.62)
**Complicated Delivery:**					
Assisted vaginal	3 (10)	5 (19.2)	3 (13.6)	1.24 (0.51–1.63)	0.91 (0.52–1.71)
Cesarean section	8 (26.7)	15 (57.7)	12 (54.5)	**1.57 (1.41–2.68)**	1.03 (0.73–1.31)
**Neonatal outcome:**					
SGA	1 (3)	1 (3.8)	2 (9)	1.52 (0.76–3.04)	**7.04 (2.36–12.42)**
Apgar < 7 at 5 min.	2 (6.6)	4 (15.3)	4 (18.2)	1.28 (0.21–1.25)	1.62 (1.15–2.53)
Neonatal death	1 (3)	2 (7.6)	1 (4.5)	1.21 (0.83–2.03)	1.13 (0.41–1.36)

* Adjusted risk ratio computed using Poisson regression with robust standard errors for: Maternal age, Race (white), SES (high), BMI, Parity, and previous adverse pregnancy outcome (preeclampsia, preterm, Gestational Diabetes); a OR * 1 = Adjusted risk ratio of PsA group; a OR * 2 = Adjusted risk ratio of AS group; SGA = small for gestational age

## Discussion

### Main findings

This study was conducted to estimate the birth outcome in pregnant women with AS and PsA and the value of the IL-17A assay. Our study showed that the use of CS was related in combination with premature delivery of SGA and oligohydramnios to high levels of IL-17A in the 3rd trimester, in pregnant women with AS and PsA.

### Strengths and limitations

The main limitation of study was the small size of study groups, and the factors strengthen the study, the prospective nature of study and use of inclusion and exclusion criteria with coding of the data collected for proper interpretation.

### Interpretation

Our findings are consistent with previous chronic inflammatory disorder studies. Females with IBD and RA are at greater risk for both premature baby and SGA infants [15, 16]. A new report was carried out by the OTIS group and showed that corticosteroid usage and a high disease activity might contribute to an elevated risk for premature birth to RA [17].

This study showed that there could be comparable tendencies in PsA and AS as the active disease increased, the risk of PsA preterm birth later in pregnancy and corticosteroid usage increased the risk of AS pre-term birth in the second trimester. On the other hand, Broms et.al [18] revealed that women with PsA have not shown a higher risk of premature birth. Various exposure criteria or different demographic variables might explain this disparity [19].

We also identified an elevated risk for C.S. in PsA, not described by the activity of illness. This result is consistent with other studies on PsA, [20] AS, [21] juvenile idiopathic arthritis [22] and inflammatory chronic arthritis [16].

We discovered an overall higher risk of C. S among the study groups over vaginal delivery, especially with previous history of spondylarthritis, sacroiliitis and hip arthritis. We discovered that elevated illness activity in the third trimester was linked to an elevated risk in this group for C. S, Also, Jakobsson et al. noticed an elevated risk of C. S amongst AS pregnant women [23, 24].

Co-morbidities including obesity could influence the risk of preterm birth and cesarean deliveries. However, after restricting the analysis to those without inflammatory bowel disease (IBD), AS or RA, the findings remained substantially unchanged. An association between obesity, including metabolic syndrome, and PsA is well established [25, 26]. Our investigation also showed that women with PsA had a higher risk of oligohydramnios. While it is unclear how this works, it is possible that the medicine she is taking and other maternal variables and/or additional comorbidities not measured or accounted for could be to blame. There was no substantial effect on the newborn outcomes for AS and PsA.

In particular, IL-17A values were shown to be elevated in the 2nd and 3rd trimesters of pregnancy and to be substantially linked with unfavorable maternal and fetal outcomes. In certain cases, IL-17 has been detected with conflicting findings in serum and in plasma samples from cases with preeclampsia. Additional studies of homeostasis between the production of the regulatory T cells by Foxp3 and CD4+ T cells produced by IL-17 might be crucial to the maternal tolerance of the semi-allogeneic fetus. Foxp3 was also decreased and IL-17A was elevated, which were related to maternal and fetal problems [27, 28].

In conclusion the majority of females with PsA and AS had an uneventful pregnancy with regard to deleterious effects, however in comparison to normal pregnancies with high levels of IL-17A in 3rd trimester, we found an elevated risk of premature birth, oligohydramnios and caesarean delivery. Future research for indications for surgical deliveries and medically indicated premature delivery, with PsA or AS knowledge is requested and pregnancy outcome will be further enhanced. Further large Studies on the influence of the illness severity and elevated IL-17A on pregnancy outcomes are also needed.

## Data Availability

The data analyzed during the current study are available from the corresponding author and can be accessed upon reasonable request.

## Abbreviations

PsA: Psoriatic arthritis
AS: Ankylosing spondylitis
IL: Interleukin
BPD: Biparietal diameter
HC: Head circumference
AC: Abdominal circumference
FL: Femur length
EFW: Estimated fetal weight
SGA: Small for gestational age
C.S.: Cesarean delivery
RAPID3: Routine Assessment of Patient Index Data 3
HAQ: Health Assessment Questionnaire
IBD: Inflammatory bowel disease
RA: Rheumatoid arthritis
ESR: Erythrocyte sedimentation rate
CRP: C-reactive protein
NSAIDs: Nonsteroidal anti-inflammatory medicines
TNF: Inhibitors of tumor necrosis factor
DMARDs: Disease-modifying antirheumatic drugs
aRR: Adjusted rate ratio
CI: Confidence interval
BMI: Body mass index
OTIS: Organization of Teratology Information Specialists.
